# Supplementary material for: Causal Relationship between Aging and Anorexia Nervosa: A White-Matter-Microstructure-Mediated Mendelian Randomization Analysis
Source: Biomedicines. 2024 Aug 16;12(8):1874. doi: 10.3390/biomedicines12081874 (PMC11351342; doi:10.3390/biomedicines12081874)
Supplement: Supplementary file 1 [file biomedicines-12-01874-s001.zip › Supplementary Figure/Figure S8/Leaveoneout plot of MR analysis for TL to ubm-b-2040.pdf]

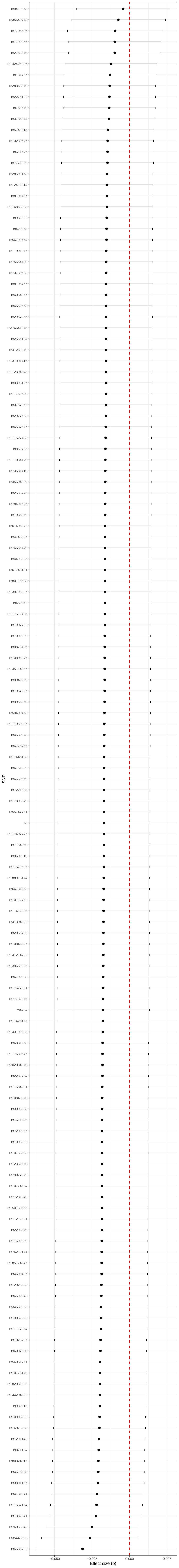
rs9419958
rs35640778
rs7705526
rs7790856
rs2763979
rs142426306
rs131797
rs28363070
rs2276182
rs762679
rs3785074
rs5742915
rs13230646
rs611646
rs7772289
rs28502153
rs12412214
rs8102497
rs116863223
rs932002
rs429358
rs56799554
rs11991877
rs75664430
rs73730598
rs8105767
rs6054257
rs6669563
rs2967355
rs376641875
rs2555104
rs41269079
rs137901416
rs112394943
rs9398196
rs11769630
rs3767952
rs2977608
rs6587577
rs111527438
rs869785
rs117034449
rs73581419
rs45604339
rs2538745
rs78491606
rs1985369
rs61405042
rs4743037
rs76666449
rs4498805
rs61748181
rs80116508
rs139795227
rs450962
rs117512405
rs1907702
rs7099229
rs9878436
rs10805346
rs145114957
rs9940099
rs1957937
rs9955360
rs59409453
rs111950327
rs4530278
rs6776756
rs17445108
rs6751209
rs6659669
rs7221585
rs17803849
rs55747751
All
rs117407747
rs7164950
rs9600019
rs11579626
rs188918174
rs66731853
rs10112752
rs11412296
rs41304832
rs2056726
rs10845387
rs141214782
rs139669835
rs6790988
rs17677991
rs77732866
rs4724
rs11426156
rs143190905
rs6881568
rs117630647
rs202034370
rs2282764
rs11584821
rs10840270
rs3093888
rs1611236
rs7209057
rs1003322
rs10768683
rs12369950
rs79977579
rs10774624
rs77231040
rs150150565
rs11212631
rs2293579
rs11699829
rs76219171
rs185174247
rs4695407
rs12925933
rs6590343
rs34550383
rs13062095
rs11117354
rs1023767
rs6007020
rs56061761
rs10773176
rs182059586
rs144204502
rs939916
rs10905255
rs16978028
rs1291143
rs871134
rs80324517
rs4616688
rs3891167
rs4731541
rs11557154
rs1332941
rs76065543
rs35446936
rs6536702
SNP
−0.050
−0.025
0.000
0.025
Effect size (b)
